# Biological Macromolecule Hydrogel Based on Recombinant Type I Collagen/Chitosan Scaffold to Accelerate Full-Thickness Healing of Skin Wounds

**DOI:** 10.3390/polym15193919

**Published:** 2023-09-28

**Authors:** Duo Kang, Wenhai Wang, Yanmei Li, Yi Ma, Yadong Huang, Jufang Wang

**Affiliations:** 1 School of Biology and Biological Engineering, South China University of Technology, Guangzhou 510006, China; 17315762692@163.com (D.K.); wangwenhai1102@163.com (W.W.); 202010108442@mail.scut.edu.cn (Y.L.); bimayikobe@scut.edu.cn (Y.M.); 2 Guangdong Provincial Key Laboratory of Fermentation and Enzyme Engineering, South China University of Technology, Guangzhou 510006, China; 3 Department of Cell Biology, Jinan University, Guangzhou 510632, China; tydhuang@jnu.edu.cn

**Keywords:** recombinant collagen, biological macromolecule hydrogels, multifunctional hydrogels, wound healing, metal–polyphenol structure

## Abstract

The development of biological macromolecule hydrogel dressings with fatigue resistance, sufficient mechanical strength, and versatility in clinical treatment is critical for accelerating full-thickness healing of skin wounds. Therefore, in this study, multifunctional, biological macromolecule hydrogels based on a recombinant type I collagen/chitosan scaffold incorporated with a metal–polyphenol structure were fabricated to accelerate wound healing. The resulting biological macromolecule hydrogel possesses sufficient mechanical strength, fatigue resistance, and healing properties, including antibacterial, antioxygenic, self-healing, vascularization, hemostatic, and adhesive abilities. Chitosan and recombinant type I collagen formed the scaffold network, which was the first covalent crosslinking network of the hydrogel. The second physical crosslinking network comprised the coordination of a metal–polyphenol structure, i.e., Cu^2+^ with the catechol group of dopamine methacrylamide (DMA) and stacking of DMA benzene rings. Double-crosslinked networks are interspersed and intertwined in the hydrogel to reduce the mechanical strength and increase its fatigue resistance, making it more suitable for clinical applications. Moreover, the biological macromolecule hydrogel can continuously release Cu^2+^, which provides strong antibacterial and vascularization properties. An in vivo full-thickness skin defect model confirmed that multifunctional, biological macromolecule hydrogels based on a recombinant type I collagen/chitosan scaffold incorporated with a metal–polyphenol structure can facilitate the formation of granulation tissue and collagen deposition for a short period to promote wound healing. This study highlights that this biological macromolecule hydrogel is a promising acute wound-healing dressing for biomedical applications.

## 1. Introduction

The skin, the largest organ of the human body, is the first line of defense against pathogen invasion [1] and is an integral part of the human immune system [2]. However, physical, chemical, thermal, mechanical, infection, and disease factors commonly contribute to deep skin injuries of various sizes [3] that cannot be repaired quickly and are vulnerable to bacterial infection. Prior to the 20th century, traditional dressings with coverage and defense functions played an essential role in wound healing [4,5], but they lacked antibacterial, antioxidant, and other effects necessary for wound healing. Moreover, they can even adhere to new granulation tissue present in the wound, thereby inducing secondary damage [3,6]. At present, biological macromolecule hydrogels embedded in various types of wound dressings are deemed promising candidates because of their exceptional advantages [7]. Biological macromolecule hydrogels exhibit excellent biocompatibility, adhesion, wettability, and air permeability [8], factors that provide a suitable microenvironment for the wound [9]. Moreover, biological macromolecule hydrogels formed from natural collagen show inherent bioactivity, excellent biocompatibility, and adhesiveness and are therefore useful for dressings designed to promote wound healing [10,11,12].

Collagen, an essential component of the extracellular matrix, is a naturally bioactive macromolecule material used in hydrogels and is mostly derived from animal skin, hooves, and cartilage [13,14,15,16,17]. However, the inferior quality, low purity, high cross-infection risk, and intense immunogenicity of animal-derived collagen can affect its performance and restrict its clinical application for biomaterials [18,19,20]. In contrast, recombinant collagen prepared via biological fermentation is a more reliable, predictable, and valuable material for constructing biomaterials because of its high quality, homogeneity, lack of virus potential, and processability [21,22,23]. Therefore, high-molecular-weight (i.e., 120 kDa) recombinant type I collagen (rCol) with good biocompatibility and satisfactory adhesiveness has been synthesized using an *E. coli* expression system for use by our lab in biomaterial construction.

Chitosan (CS) is a natural macromolecule compound extracted from chitin that possesses cytocompatibility, biocompatibility, cell attachment/proliferation/migration, antibacterial properties, and mechanical stability [24,25,26,27]. Currently, chitosan-based bioinks are widely used for bone, cartilage, vascular system, neural network, and skin tissue regeneration. In addition, chitosan can absorb organic pollutants and heavy metal ions to fulfill the needs of environmental purification and drug delivery [27]. However, its poor mechanical performance and low solubility are major factors that limit its application [28,29]. To solve these problems, chitosan and collagen have been blended to form a biological macromolecule hydrogel [30]. This hydrogel system has shown potential for cell encapsulation and can stimulate stem cell differentiation and angiogenesis in vivo [31]. In these systems, collagen and chitosan hydrogels are synthesized without covalent bonding, which essentially limits their mechanical strength. Therefore, Deng et al. introduced the crosslinked compound 1-(3-(Dimethylamino) propyl)-3-ethylcarbodi-imidehydrochloride (EDC) to enhance the strength and bioactivity of collagen and chitosan hydrogels [32]. However, unreacted EDC in the hydrogel is difficult to remove and may induce cytotoxicity. Therefore, a double bond (i.e., via a methacrylic group) was introduced onto the side-chain amino acids of chitosan and collagen for free radical polymerization via a photo-initiator under *UV* light [33,34]. Photo-crosslinking approaches have been widely used owing to their spatiotemporal controllability, fast speed, and low rates of cellular death [35].

Copper ions are a promising antimicrobial agent. It was shown that samples with moderate amounts of copper can be used in future tissue engineering because this amount of copper is non-toxic to cells and inhibits the growth of antibiotic-resistant bacteria at the same time [36,37]. In addition, copper nanoparticles are commonly added to hydrogels to enhance their antibacterial properties; however, several studies have pointed out that the addition of metal nanoparticles to hydrogels alone may lead to poor dispersion and slow degradation [38,39]. One approach to avoid these problems is to introduce a metal and polyphenol, i.e., Cu^2+^ and dopamine methacrylamide (DMA), into hydrogels simultaneously. The catechol group in DMA can form a stable complex with Cu^2+^ [40]. Furthermore, the hydroxy group of DMA can interact with amino or sulfhydryl groups within tissue molecules [41], resulting in tissue adhesion and antioxygenic properties [42,43,44].

**Figure 1:**
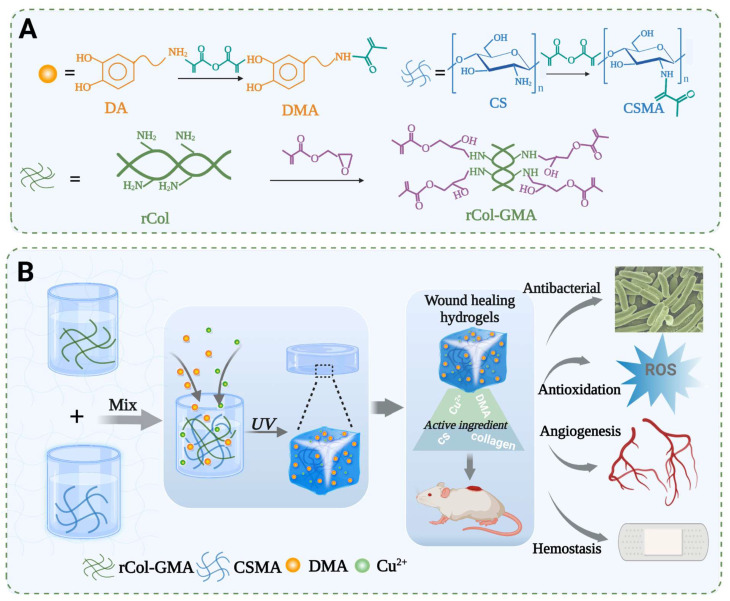
(**A**) The synthesis of chitosan methacrylate (CSMA), glycidyl methacrylate recombinant collagen (rCol-GMA), and dopamine methacrylamide (DMA). (**B**) Schematic representation of the formation of the chitosan methacrylate (CSMA)/glycidyl methacrylate recombinant collagen (rCol-GMA)/copper ions (Cu^2+^)/dopamine methacrylamide (DMA) (abbreviation: CS/rCol/Cu/DMA) hydrogel and its applications for wound healing.

In this study, we used the photo-crosslinking method to prepare a multifunctional, biological macromolecule hydrogel based on a recombinant type I collagen/chitosan scaffold incorporated with a metal–polyphenol structure. This biological macromolecule hydrogel possesses self-healing, antioxygenic, and adhesive abilities, as well as antibacterial, vascularization, and hemostatic properties, and can therefore effectively accelerate wound healing (Figure 1B). To obtain this multifunctional, biological macromolecule hydrogel, methacrylic groups were grafted onto side-chain amino groups of rCol and chitosan to form the scaffold, i.e., the first master photo-crosslinking network. Next, we added Cu^2+^ and DMA to the scaffold to construct a double-crosslinked network structure for the completed hydrogel (i.e., resulting in a CS/rCol/Cu/DMA hydrogel). The rheological, mechanical, and adhesion properties of the hydrogel were then characterized. In addition, the biocompatibility, antibacterial, hemostatic, antioxidant, and vascularization abilities of the biological macromolecule hydrogel were also tested. Finally, the ability of the hydrogel to promote vascular regeneration and collagen deposition was explored during wound healing using a full-thickness skin defect model. Taken together, *UV* irradiation can turn the hydrogel precursor solution into a gel, and it also has the function of disinfection and sterilization [45]. Our final results indicated that this biological macromolecule hydrogel has many desirable functional properties and therefore may be broadly applicable for applications related to wound healing.

## 2. Methods

### 2.1. The Synthesis of CS/rCol/Cu/DMA Hydrogels

The specific processes for synthesizing glycidyl methacrylate recombinant collagen (rCol-GMA), chitosan methacrylate (CSMA), and dopamine methacrylamide (DMA) are reported in the Supplementary Materials. First, CSMA, rCol-GMA, DMA, copper chloride (CuCl_2_), and lithium phenyl-2,4,6-trimethylbenzoylphosphinate (LAP) were dissolved at concentrations of 4 wt%, 4 wt%, 8.225 wt%, 10 wt%, and 1 wt%, respectively. The molar ratio of Cu^2+^ to DMA was set to 1:2. The Cu^2+^ final concentrations of Cu_50_, Cu_100_, and Cu_200_ were 50 μg mL^−1^ (0.5 μL), 100 μg mL^−1^ (1 μL), and 200 μg mL^−1^ (2 μL), respectively. The DMA volumes corresponding to Cu^2+^ treatments were 2 μL, 4 μL, and 8 μL (Table S1, Supplementary Materials). Next, CSMA (400 μL) and rCol-GMA (400 μL) solutions were mixed, and Cu^2+^ and DMA were added at fixed proportions. The final concentration of 0.05 wt% LAP was then added to the mixed solution listed above for agitation. In addition, DI water was used to bring the volume of the hydrogel precursor solution to 1 mL. The hydrogel precursor solution was then placed under *UV* light (i.e., 365 nm, 1 w cm^−2^) for 15 s, and the sol–gel transition was completed.

### 2.2. Rheology of CS/rCol/Cu/DMA Hydrogels

A TA rheometer (HR30, WATERS, Newcastle, DE, USA) was used to measure the rheological behavior, shear-thinning, and self-healing properties of the hydrogel. These details are shown in the Supplementary Materials.

### 2.3. Compression Test of CS/rCol/Cu/DMA Hydrogels

A compression test and cyclic compression tests were performed according to a previously published method [46]. The specific processes used are represented in the Supplementary Materials.

### 2.4. In Vitro Contact Antibacterial Activity of CS/rCol/Cu/DMA Hydrogels

Next, the antibacterial activity of the hydrogel was evaluated using a contact antibacterial test. *E. coli* DH5α and *S. aureus* (ATCC 29213) were employed for testing the hydrogel surface antibacterial activity using a method published in a previous study [47]. First, the hydrogel (400 μL) was added to a 48-well plate, and 10 μL of bacterial suspension (10^6^ colony-forming units (CFUs) mL^−1^) was added onto its surface. Next, the 48-well plate was placed in an incubator at 37 °C for 2 h. Then, the surviving bacteria on the 48-well plate were resuspended with phosphate-buffered saline (PBS). The resuspended bacterial (10 μL) solution was applied to a Luria–Bertani (LB) plate, and 10 μL of a bacterial suspension (10^6^ CFUs mL^−1^) was used as a control. Finally, the above LB plates were incubated for 18–24 h at 37 °C, and the CFUs on the Petri dish were counted. This test was repeated three times, and the results were expressed as the bacterial survival ratio (%) as per the following equation:

(1) $$\mathrm{Bacterial}\;\mathrm{survival}\;\mathrm{ratio}\;(\%)=\frac{\;\mathrm{surviving}\;\mathrm{bacterial}\;\mathrm{count}\;\mathrm{of}\;\mathrm{the}\;\mathrm{experimental}\;\mathrm{group}}{\mathrm{bacteria}\;\mathrm{count}\;\mathrm{of}\;\mathrm{the}\;\mathrm{control}}\;\times \;100\%$$

### 2.5. ROS Scavenging Ability and Angiogenesis Capacity

The antioxidant capacity and angiogenesis properties of the hydrogel were tested according to previously published methods [48,49]. The details of these tests are reported in the Supplementary Materials.

### 2.6. In Vivo Full-Thickness Wound-Healing Test

All animal protocols were reviewed and approved by the Institutional Animal Care and Use Committee of the South China University of Technology (Approval No. 2019053). A full-thickness skin wound model was created in standard deviation (SD) rats (i.e., 4–6 weeks, 180–220 g, female) according to a previously published protocol [50]. All wounds were photographed on days 0, 3, 7, 14, and 21, respectively, and wound boundaries were drawn using Image J 1.52v (Wayne Rasband National Institutes of Health, Bethesda, MD, USA) [51]. The wound closure rate was calculated as follows:

(2) $$\mathrm{Wound}\;\mathrm{closure}\;\mathrm{ratio}\;\left(\%\right)=\frac{(\mathrm{Original}\;\mathrm{wound}\;\mathrm{area}\;-\;\mathrm{Open}\;\mathrm{area}\;\mathrm{on}\;\mathrm{the}\;\mathrm{indicated}\;\mathrm{day})}{\mathrm{Original}\;\mathrm{wound}\;\mathrm{area}}\times \;100\%$$

Histological and immunohistochemistry examinations were performed to assess vascular and skin remodeling during wound healing. CD31 was selected for immunohistochemistry staining [52]. The specific protocols used are provided in the Supplementary Materials.

### 2.7. Statistical Analyses

All experiments were replicated at least three times, and results were expressed as mean ± SD. Statistical analysis of all datasets was performed using one-way analysis of variance. *p*-values < 0.05 were considered to be statistically significant (* *p* < 0.05, ** *p* < 0.01, *** *p* < 0.001).

## 3. Results and Discussion

### 3.1. Acquisition of Recombinant Type I Collagen

Collagen, as a natural biological macromolecule hydrogel material with high biocompatibility and degradability, has been used extensively for bioengineering applications [20]. In this study, rCol was obtained via molecular cloning and by use of an *E. coli* expression system (Figure S2A, Supplementary Materials). Subsequently, a Western blot was used to validate the expression of rCol. As shown in Figure S1 (Supplementary Materials), the molecular weight of rCol was 120 kDa, and rCol was expressed in the supernatant. After purification using Ni column affinity chromatography, the purity of rCol was greater than 90% (Figure S2B, Supplementary Materials). The secondary structure of rCol was then examined via circular dichroism spectroscopy (CD). As shown in Figure S2C (Supplementary Materials), the circular dichroism of rCol in the far ultraviolet region (i.e., 190–250 nm) exhibited a negative peak at approximately 198 nm and a positive peak at 215 nm. This was similar to the characteristic circular dichroism of natural collagen, which shows a negative absorption peak at 197 nm and a weak positive absorption peak at 220 nm [53]. Cellular assays revealed that NIH/3T3 cells cultured in rCol promoted cell proliferation and adhesion, demonstrating high cytocompatibility and satisfactory adhesion compared with a control group. Taken together, these results indicate that this material has strong potential for constructing multifunctional, biological macromolecule hydrogels (Figures S3 and S4, Supplementary Materials).

### 3.2. Preparation of CS/rCol/Cu/DMA Hydrogels

**Figure 2:**
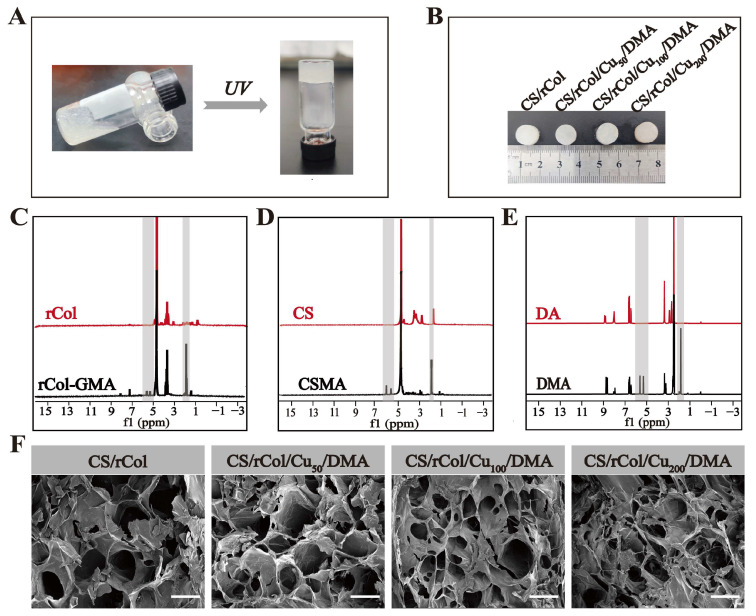
(**A**) Photographs of the biological macromolecule hydrogel before and after photo-crosslinking. (**B**) Photograph of the chitosan methacrylate (CSMA)/glycidyl methacrylate recombinant collagen (rCol-GMA) (abbreviation: CS/rCol), chitosan methacrylate (CSMA)/glycidyl methacrylate recombinant collagen (rCol-GMA)/copper ions (50 μg/mL Cu^2+^)/dopamine methacrylamide (DMA) (abbreviation: CS/rCol/Cu_50_/DMA), chitosan methacrylate(CSMA)/glycidyl methacrylate recombinant collagen (rCol-GMA)/copper ions (100 μg/mL Cu^2+^)/dopamine methacrylamide (DMA) (abbreviation: CS/rCol/Cu_100_/DMA), and chitosan methacrylate(CSMA)/glycidyl methacrylate recombinant collagen (rCol-GMA)/copper ions (200 μg/mL Cu^2+^)/dopamine methacrylamide (DMA) (abbreviation: CS/rCol/Cu_200_/DMA) hydrogels. ^1^H-NMR spectra of (**C**) recombinant collagen (rCol) and rCol-GMA; (**D**) chitosan (CS) and CSMA; (**E**) dopamine (DA) and DMA. (**F**) Scanning electron microscopy (SEM) images of the CS/rCol/Cu/DMA hydrogels (scale bars = 250 µm).

Although recombinant collagen has excellent biocompatibility, pure collagen hydrogels lack sufficient mechanical strength and show minimal self-healing, antibacterial, or antioxygenic properties. These factors hinder its practical applicability for use in clinical treatments. Here, the mechanical properties were enhanced by complexing recombinant type I collagen with chitosan. First, the grafting of methacrylic anhydride and glycidyl methacrylate on the side-chain amino groups of chitosan and rCol, respectively, facilitated their formation of photolinking networks (Figure 1A). Dopamine methacrylamide (DMA) was also synthesized to improve the adhesion and antioxidant capacity of the hydrogel (Figure 1A). The CS/rCol/Cu/DMA hydrogel precursor solution was obtained by mixing rCol-GMA, CSMA, Cu^2+^, and DMA solutions. Next, a series of photolinking, biological macromolecule hydrogels (i.e., CS/rCol, CS/rCol/Cu_50_/DMA, CS/rCol/Cu_100_/DMA, and CS/rCol/Cu_200_/DMA), each with different morphological and mechanical properties, were formed under *UV* light (Figure 2A,B).

### 3.3. Characterization of CS/rCol/Cu/DMA Hydrogels

The chemical structures of rCol-GMA, CSMA, and DMA were then verified using ^1^H-NMR and FT-IR. As shown in Figure 2C, characteristic resonances of the methacrylate vinyl group (i.e., δ = 5.32 ppm and 5.62 ppm) verified the successful synthesis of rCol-GMA. In addition, characteristic resonance signals of the methacrylate group (i.e., δ = 5.62 ppm and 5.95 ppm (CSMA); δ = 5.30 ppm and 5.62 ppm (DMA)), confirmed the successful bonding of these methacrylate groups to CS and DA, respectively (Figure 2D,E). As shown in Figure S5 (Supplementary Materials), the FT-IR spectral results demonstrated that rCol-GMA has a peak of 1632 cm^−1^, which corresponds to the C=O stretching vibration (also known as the amide I band). Notably, rCol-GMA corresponded to CHOH stretching of the alcohol group at 1384 cm^−1^, which can be attributed to the opening of an epoxy group of GMA (Figure S5, Supplementary Materials). The characteristic peak at 1650 cm^−1^ was generated by the stretching vibration of C=O in the amide group, indicating that amination occurred during DMA synthesis [54], and a characteristic peak near 3225 cm^−1^ demonstrated the stretching vibration of the OH in the catechol group (Figure S6, Supplementary Materials). Similarly, for CSMA, peaks at 1701 cm^−1^, 1652 cm^−1^, and 837 cm^−1^ suggested the presence of a C=O group and a C=C group (Figure S7, Supplementary Materials). Taken together, these findings confirmed that a vinyl group was successfully grafted onto the side-chain amino groups of rCol, CS, and DA.

In general, the porous network of a hydrogel can deliver nutrients and oxygen while also assisting in maintaining the moisture content of the surrounding environment. The microstructure of CS/rCol/Cu/DMA hydrogels was then observed using scanning electron microscopy (SEM; FEI NOVA NANO450, Columbia, MD, USA). As shown in Figure 2F, these hydrogels exhibited an interconnected 3D porous network structure. The porosities of CS/rCol, CS/rCol/Cu_50_/DMA, CS/rCol/Cu_100_/DMA, and CS/rCol/Cu_200_/DMA calculated via ImageJ for each sample type were 33.54%, 35.58%, 38.40%, and 44.13%, respectively. In addition, the pore size decreased with increased concentrations of DMA and Cu^2+^. This may be due to π–-π interactions within DMA and/or the interaction between the catechol group of DMAs and Cu^2+^, which enhances the crosslinking of the hydrogel.

**Figure 3:**
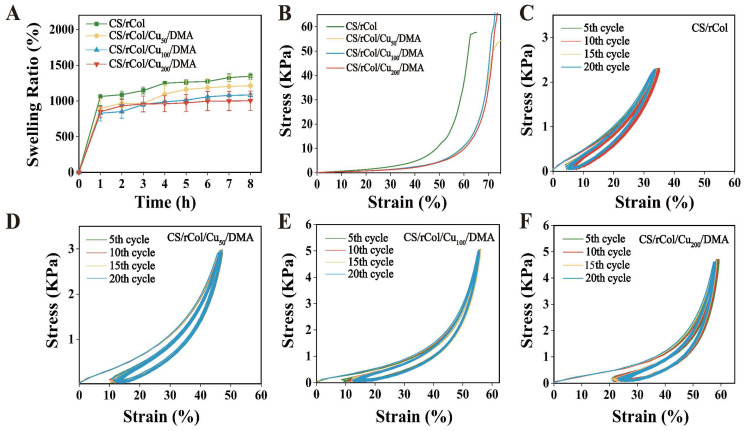
(**A**) Swelling ratio of CS/rCol, CS/rCol/Cu_50_/DMA, CS/rCol/Cu_100_/DMA, and CS/rCol/Cu_200_/DMA hydrogels (*n* = 3). (**B**) Mechanical properties of CS/rCol, CS/rCol/Cu_50_/DMA, CS/rCol/Cu_100_/DMA, and CS/rCol/Cu_200_/DMA hydrogels. Cyclic compression curves for (**C**) the CS/rCol hydrogel, (**D**) the CS/rCol/Cu_50_/DMA hydrogel, (**E**) the CS/rCol/Cu_100_/DMA hydrogel, and (**F**) the CS/rCol/Cu_200_/DMA hydrogel.

Skin damage causes wound sites to overproduce secretions, which can induce bacterial growth and delay wound healing. Therefore, hydrogels should possess an appropriate swelling ability to absorb excessive exudate produced by the wound [8,54]. Here, the swelling rate of the hydrogel was evaluated using PBS. As shown in Figure 3A, all hydrogels swelled rapidly in PBS, confirming that they showed robust water absorption abilities. Furthermore, CS/rCol/Cu/DMA hydrogels reached a swelling equilibrium rapidly (i.e., within 4 h), while the CS/rCol hydrogel only started to reach a swelling equilibrium by 4 h; this indicated that the CS/rCol/Cu_50_/DMA, CS/rCol/Cu_100_/DMA, and CS/rCol/Cu_200_/DMA hydrogels showed a lower degree of swelling than the CS/rCol hydrogel. These results can be explained by the addition of Cu^2+^ and DMA to the hydrogel, which enhances hydrogel crosslinking, shortens the intermolecular gap, and reduces pore size.

Ideal hydrogel dressings should possess mechanical properties that can withstand environmental changes. Here, the mechanical properties of the hydrogel were measured using a dynamic thermomechanical analyzer. As shown in the stress–strain curve of hydrogel in Figure 3B, the CS/rCol hydrogel fractured under a strain of 60% (i.e., the green curve), whereas the CS/rCol/Cu_50_/DMA, CS/rCol/Cu_100_/DMA, and CS/rCol/Cu_200_/DMA hydrogels could withstand strains of >70% without fracturing (i.e., the red, blue, and yellow curves). Compressive modulus plots of the hydrogels were obtained by selecting the interval of 5–25% strain in the above stress–strain curve. The compression modulus of the hydrogel showed that the CS/rCol hydrogel had higher mechanical strength than the CS/rCol/Cu_50_/DMA, CS/rCol/Cu_100_/DMA, and CS/rCol/Cu_200_/DMA hydrogels (Figure S8, Supplementary Materials). These data indicate that a single CS/rCol network cannot withstand large strains, but the introduction of the metal–polyphenol structure to form a dual network increases strain resistance significantly. The metal–polyphenol structure reduced the charge density of the entire network, enabling it to interconnect and intertwine so that the pressure could be uniformly dispersed to other networks. To further verify the fatigue resistance results, the hydrogel was again analyzed using a dynamic thermomechanical analyzer. As shown in Figure 3C–F, cyclic compression curves showed that hydrogels with Cu^2+^ and DMA showed no significant damage when strains greater than 40% were applied. They also exhibited a small hysteresis loop, indicating that the biological macromolecule hydrogel had low energy dissipation. These results suggest that the hydrogel was highly resistant to environmental strain, a property that permits them to cope with higher strain requirements and demanding environments. In short, the CS/rCol scaffold in the hydrogel provided structural strength and stability, whereas the metal–polyphenol structure (i.e., Cu–DMA) provided adhesion and fatigue resistance by interacting with the CS/rCol scaffold. Thus, the strategy of designing a biological macromolecule hydrogel is a suitable option for resolving the balance between mechanical strength and resistance.

### 3.4. Rheological, Shear-Thinning, and Self-Healing Property Tests

**Figure 4:**
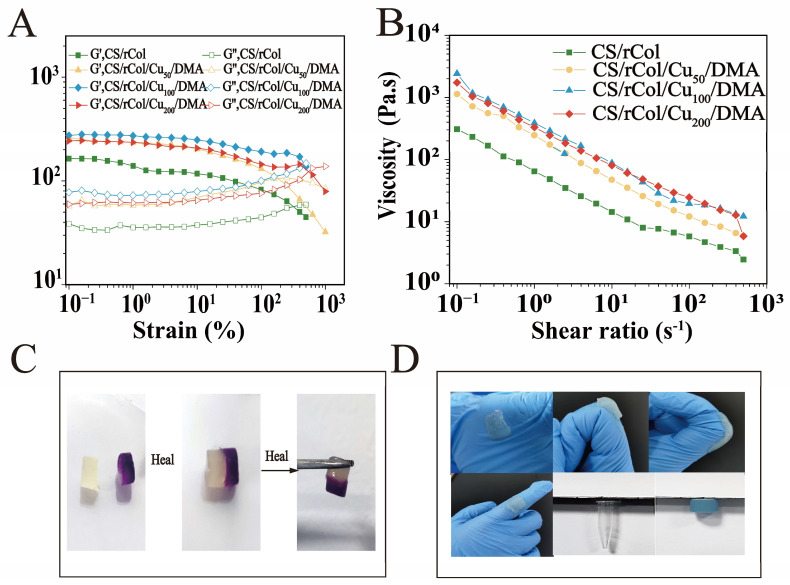
(**A**) Rheological test results for CS/rCol, CS/rCol/Cu_50_/DMA, CS/rCol/Cu_100_/DMA, and CS/rCol/Cu_200_/DMA hydrogels. (**B**) Shear-thinning of the CS/rCol, CS/rCol/Cu_50_/DMA, CS/rCol/Cu_100_/DMA, and CS/rCol/Cu_200_/DMA hydrogels. (**C**) Photographs of the self-healing properties of the CS/rCol/Cu_100_/DMA hydrogels. (**D**) Photographs of CS/rCol/Cu_100_/DMA hydrogel adhesive properties.

We found that the internal structural changes of the hydrogel were closely related to its rheological properties. A rheometer was used to examine these rheological properties. The storage modulus (G′) and loss modulus (G″) of various hydrogels were measured at a fixed frequency (10 rad s^−1^). For the strain sweep shown in Figure 4A, the G′ and G″ values of the hydrogel fluctuated slightly before strain = 100%, and the G’ value was higher than the G″ value, implying that the dual-network hydrogel had gel characteristics and the dual network was relatively stable; i.e., it could not be destroyed easily even under severe strain. After strain = 100%, the G′ value of the dual-network hydrogel was lower than the G″ value, showing that the coordination bonds had broken and the dual-network structure had collapsed.

**Figure 5:**
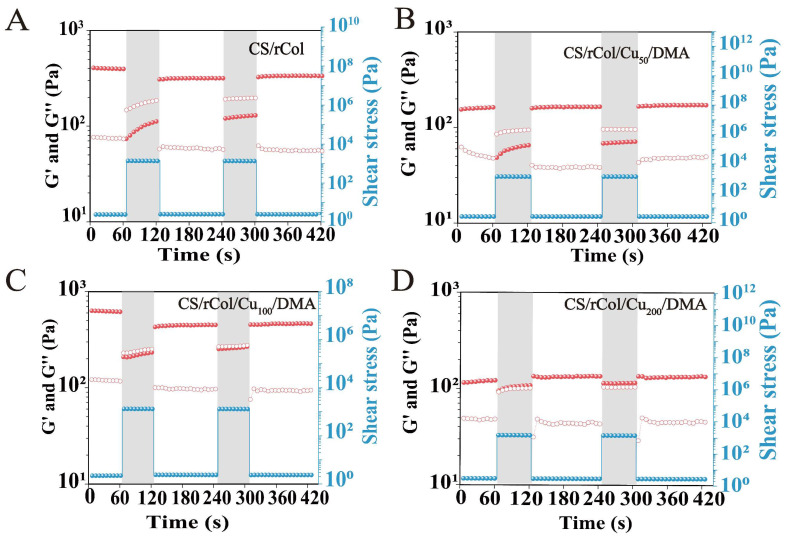
Self-healing test results for (**A**) the CS/rCol hydrogel, (**B**) the CS/rCol/Cu_50_/DMA hydrogel, (**C**) the CS/rCol/Cu_100_/DMA hydrogel, and (**D**) the CS/rCol/Cu_200_/DMA hydrogel.

Hydrogels may suffer from external mechanical stress, which can disrupt the integrity of the internal network [55,56]. Therefore, hydrogels must possess a certain degree of self-healing ability to offer sustainable wound protection. The self-healing capacity of the biological macromolecule hydrogel was therefore explored using a macroscopic healing test. As shown in Figure 4C, a cylindrical CS/rCol/Cu_100_/DMA hydrogel sample was cut and dyed. Next, the interface boundary of two pieces of semicylindrical hydrogels was blurred and recovered to its original shape after the two fractured hydrogels were combined in 5 min. Self-healing was assessed again using a rheometer to further analyze the self-healing properties of the dual-network hydrogel. As shown in Figure 5A–D, the initial G′ value of the hydrogel was higher than the G″ value, demonstrating that the hydrogel network was stable. As more pressure was applied, both moduli dropped sharply, and the G″ value was then higher than the G′ value. This result shows the destruction of the hydrogel network in response to high stress. All moduli recovered after the pressure was released, and the G′ value was once again higher than the G″ value. Notably, both G′ and G″ could return to over 90% of their initial value even after two cycles. These results confirm the exceptional self-healing potential of the biological macromolecule hydrogel tested here.

Multifunctional, biological macromolecule hydrogels can firmly adhere to the surface of a wound as physical barriers to prevent bacterial invasion and expedite wound recovery. Moreover, they do this considerably more efficiently than traditional hydrogels [57]. Here, the adhesion performance of the hydrogel was tested macroscopically using dissimilar materials. As illustrated in Figure 4D, the CS/rCol/Cu_100_/DMA hydrogel sample could adhere to joints and plastics and can adapt to various bending angles, thereby allowing the hydrogel to be used in many settings. The hydrogel shear-thinning curve tested using a rheometer is shown in Figure 4B; the viscosity of the hydrogels decreased with increasing shear rates (i.e., from 0.1 to 500 s^−1^). Moreover, the viscosities of the CS/rCol/Cu_50_/DMA, CS/rCol/Cu_100_/DMA, and CS/rCol/Cu_200_/DMA hydrogels were preferable to that of the CS/rCol hydrogel. This may have been caused by the combined effect of dopamine and collagen in the network. These results demonstrate that the biological macromolecule hydrogels showed satisfactory adhesion and were suitable for use for clinical wound treatment.

### 3.5. Contact Antibacterial Activity of the Hydrogel

**Figure 6:**
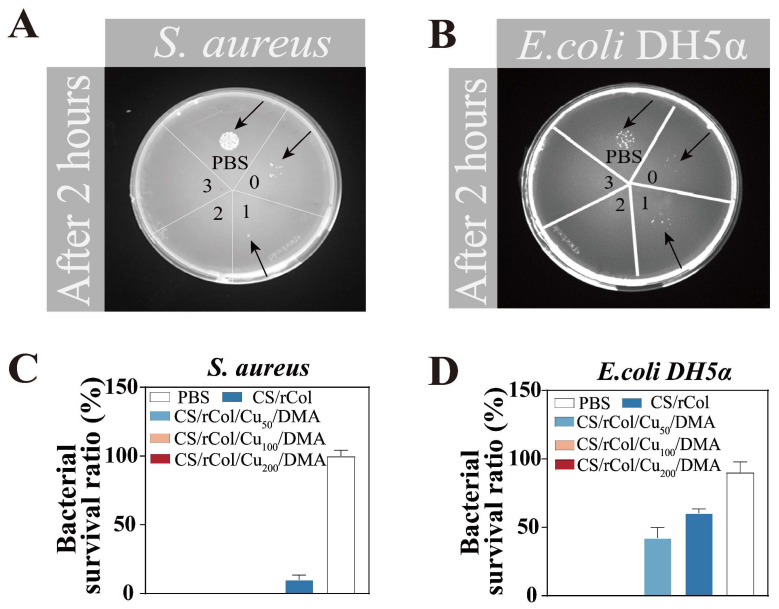
In vitro hydrogel antibacterial effect on (**A**) *Staphylococcus aureus (S. aureus)* (ATCC 29213) and (**B**) *Escherichia coli* (*E. coli)* DH5α. Shown are 0: CS/rCol hydrogel; 1: CS/rCol/Cu_50_/DMA hydrogel; 2: CS/rCol/Cu_100_/DMA hydrogel; and 3: CS/rCol/Cu_200_/DMA hydrogel (black arrow is microbial colony). Quantitative in vitro results of the hydrogel against (**C**) *S. aureus* (ATCC 29213) and (**D**) *E. coli* DH5α.

Hydrogel dressings should have specific antibacterial properties to diminish the threat of wound infection [58]. Therefore, the bacteriostatic properties of the hydrogel were assessed using the contact bacteriostatic method. As shown in Figure 6A,B, no *S. aureus* or *E. coli* DH5α growth was observed on LB plates in the areas labeled as 2 (CS/rCol/Cu_100_/DMA hydrogel) and 3 (CS/rCol/Cu_200_/DMA hydrogel). The quantitative results also showed that the CS/rCol/Cu_100_/DMA and CS/rCol/Cu_200_/DMA hydrogels had a near 100% killing effect for *S. aureus* and *E. coli* DH5α (Figure 6C,D). This may have been caused by the synergistic antibacterial effect of Cu^2+^ and chitosan in the hydrogels. Judging from the antibacterial effect against *S. aureus* and *E. coli* DH5α, the hydrogels in general demonstrated relatively low antibacterial activity against *E. coli* DH5α. For instance, only the CS/rCol/Cu_100_/DMA and CS/rCol/Cu_200_/DMA hydrogels could completely kill *E. coli* DH5α, whereas CS/rCol/Cu_50_/DMA hydrogels achieved a 100% killing effect against *S. aureus*. This weak bactericidal effect against *E. coli* DH5α may be attributed to the bilayer membrane structure of *E. coli*, which prevents antibacterial components from invading the cell interior [56]. However, for *S. aureus,* the effective antibacterial components in hydrogels can readily bind to its surface and then destroy the cell membrane to kill the host bacterium.

Next, we tested the release behavior of Cu^2+^. As shown in Figure S9 (Supplementary Materials), Cu^2+^ was rapidly released from hydrogels within 1 h. At this point, the Cu^2+^ release rate began to slow down until it reached sustained release after 1 h; this suggests that the hydrogel can provide a sustained antibacterial effect. From the perspective of wound healing, the Cu^2+^ initially released by the hydrogel shortly after application can help guard wounds against external pathogens at first. Then, the sustained release of Cu^2+^ can endow the hydrogel with a long-lasting antibacterial effect and stimulate the formation of blood vessels during the late stages of wound healing.

### 3.6. In Vitro Biocompatibility, Angiogenesis, and ROS Scavenging Ability

**Figure 7:**
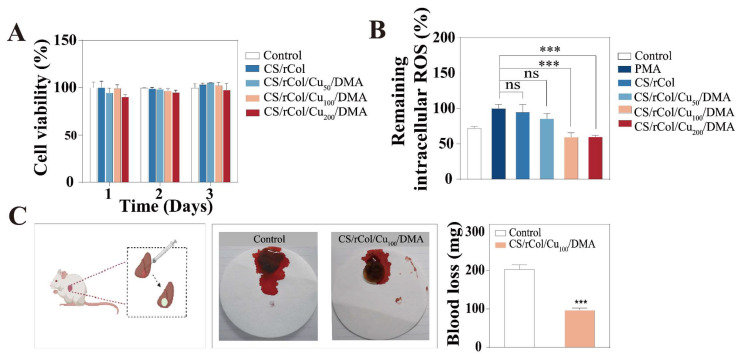
(**A**) Cell viability of human umbilical vein endothelial cells (HUVECs) cultured in a hydrogel extract solution following a CCK-8 assay (*n* = 3). (**B**) In vitro reactive oxygen species (ROS) scavenging ability of CS/rCol, CS/rCol/Cu_50_/DMA, CS/rCol/Cu_100_/DMA, and CS/rCol/Cu_200_/DMA hydrogels (*n* = 3). (**C**) Mouse liver hemorrhage model, bloodstain photographs, and quantitative mouse liver blood loss results (*n* = 3). Data represent mean ± SD; * *p* < 0.05, ** *p* < 0.01, *** *p* < 0.001, “ns” is no significant difference.

Biocompatibility is a crucial factor for the use of a hydrogel in clinical dressings [59]. The Cell Counting Kit-8 (CCK-8) assay was used for detecting cell biocompatibility. As shown in Figure 7A, the CCK-8 assay results revealed no significant differences in hydrogel-treated HUVEC viability compared with control HUVECs after being cultured in a hydrogel extract solution for 1, 2, or 3 days; this result demonstrates the high level of biocompatibility of the biological macromolecule hydrogel.

ROS scavenging properties are also required for hydrogels to facilitate wound repair [60,61]. In this work, fluorescence intensity was measured using an enzyme labeling instrument after NIH/3T3 cells were incubated with a hydrogel extract solution. According to Figure 7B, the CS/rCol/Cu_100_/DMA and CS/rCol/Cu_200_/DMA groups significantly decreased intracellular ROS relative to a phorbol ester (PMA)-treated control group. These findings revealed that the Cu^2+^ and dopamine present in the hydrogels exhibited strong antioxidant capacity, which effectively alleviated cellular oxidative stress and reduced ROS within the wound.

As reported by a previous study, Cu^2+^ can significantly accelerate wound healing by stimulating vascularization [48]. Matrigel was employed to assess the potential for vascularization promotion of hydrogels when added to HUVECs. After 4 h of induction, hydrogel groups with Cu^2+^ produced a more developed capillary network structure relative to the control group (Figure S10). Notably, the CS/rCol/Cu_100_/DMA and CS/rCol/Cu_200_/DMA hydrogel groups produced more elongated vessels than the CS/rCol/Cu_50_/DMA group. This effect is likely ascribed to the CS/rCol/Cu_100_/DMA and CS/rCol/Cu_200_/DMA networks having higher Cu^2+^ content.

### 3.7. Hemostasis Performance of Hydrogels

An ideal hydrogel should boost wound clotting via physical hemostasis [62]. Therefore, the CS/rCol/Cu_100_/DMA hydrogel with the best performance was chosen for hemostatic capacity analysis in a liver bleeding model (Figure 7C). Compared to a control group (203.33 mg), the blood loss of the CS/rCol/Cu_100_/DMA hydrogel group was significantly reduced (96.66 mg), and the hydrogel had a significant hemostatic effect during wound healing (Figure 7C). The following factors may be the reason for the hemostatic effect of the CS/rCol/Cu_100_/DMA hydrogel: First, this hydrogel tightly adhered to the skin surface to absorb blood exuding from the wound. Second, collagen and chitosan in the hydrogel could stimulate platelet adhesion and aggregation to stanch bleeding.

### 3.8. In Vivo Wound-Healing Evaluation

**Figure 8:**
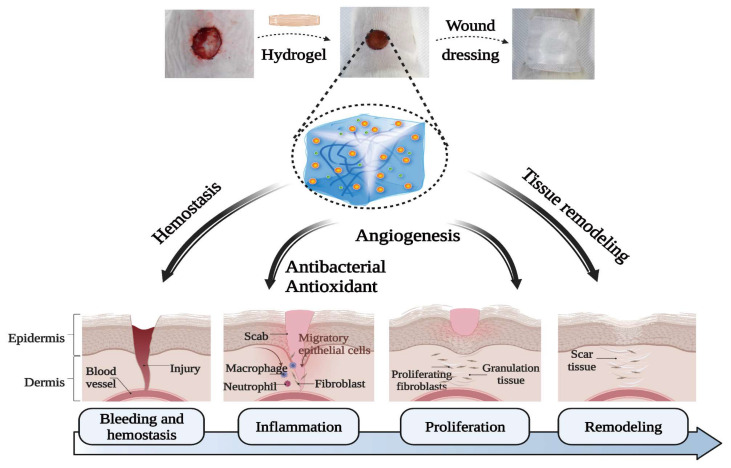
Schematic illustration of the CS/rCol/Cu_100_/DMA hydrogel and its effects on wound healing at different stages.

**Figure 9:**
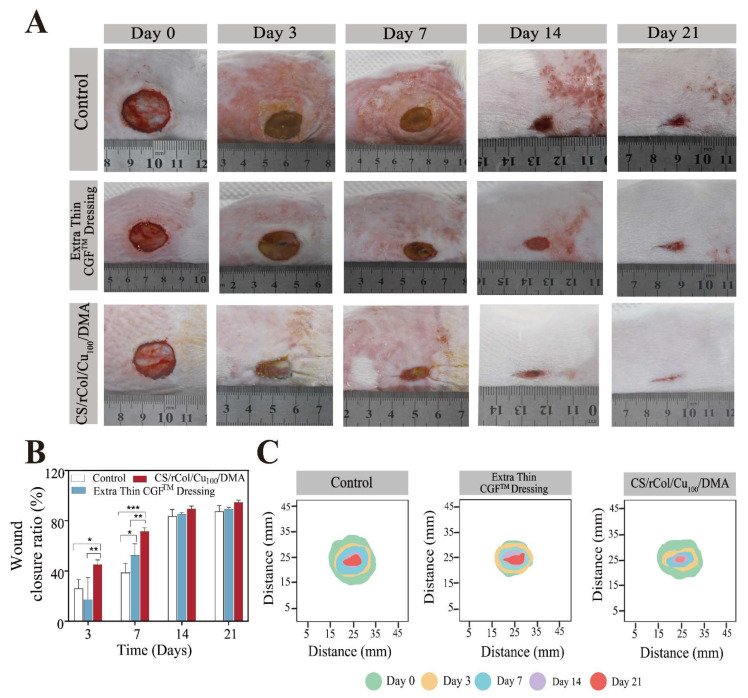
(**A**) Photographs of the wound site after 0, 3, 7, 14, and 21 days. (**B**) Mean wound closure ratio measurements over time in days (*n* = 4). (**C**) Schematic presentation of the wound site after 0, 3, 7, 14 and 21 days. Data represent mean ± SD; * *p* < 0.05, ** *p* < 0.01, *** *p* < 0.001.

The effectiveness of the CS/rCol/Cu_100_/DMA hydrogel with respect to wound healing was tested using a full-thickness skin defect model. Here, wounds were covered with CS/rCol/Cu_100_/DMA hydrogel and fixed with gauze (Figure 8). Next, the effectiveness of the hydrogel was assessed at different stages of wound healing (Figure 8). Wound size was used to intuitively assess the therapeutic efficiency of the CS/rCol/Cu_100_/DMA hydrogel group relative to the negative and positive control groups. Surprisingly, the wound size of the CS/rCol/Cu_100_/DMA hydrogel group decreased more rapidly than that of the negative and positive control groups during wound healing (Figure 9A,C). The wound closure rate of the hydrogel group was 71.4% by the 7th day; this was a faster healing rate than that of the negative control group (38.8%) or the positive control group (52.8%) by the same time point (Figure 9B). The CS/rCol/Cu_100_/DMA hydrogel was applied only at the time of wound formation (i.e., repeated treatment was not used), resulting in no gap in the wound closure rate between the control group and hydrogel-treated group by days 14 and 21. Nevertheless, the ability of CS/rCol/Cu_100_/DMA hydrogel to facilitate rapid wound recovery makes it suitable for the treatment of acute wounds.

**Figure 10:**
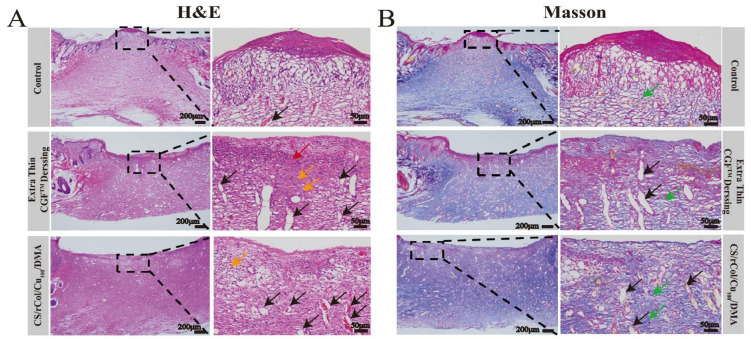
(**A**) Hematoxylin and eosin (H & E) staining images of wound tissue sections on day 21 (scale bars = 200 µm and 50 µm for inset image). Yellow arrow, fibroblast; black arrow, new vessels; red arrow, inflammatory cells. (**B**) Masson trichrome staining images of wound tissue sections on day 21 (scale bar = 200 µm and 50 µm for inset image). Green arrow, collagenous fiber; black arrow, new vessels.

**Figure 11:**
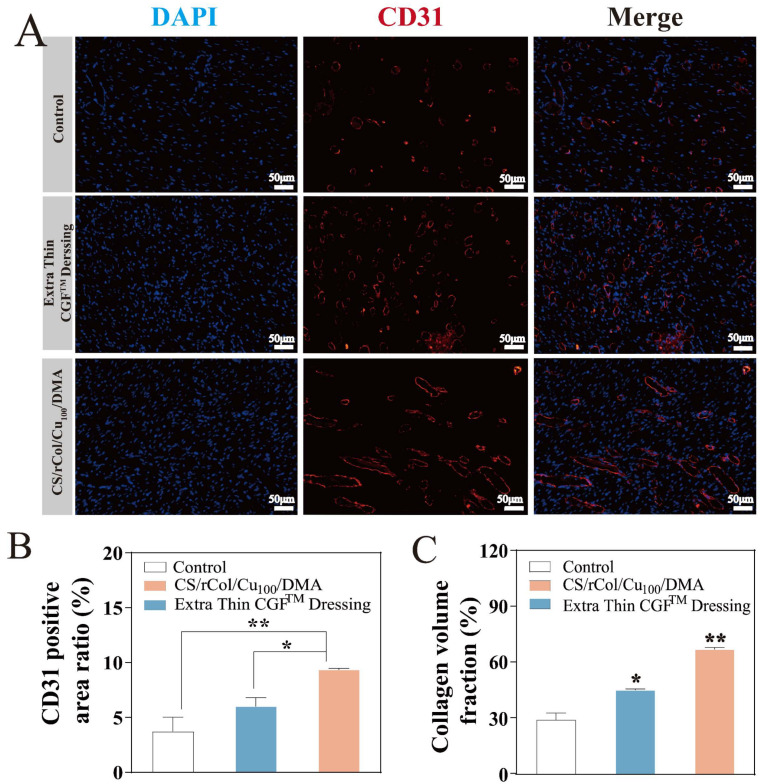
(**A**) Representative photographs of immunofluorescence staining of platelet endothelial cell adhesion molecule-1 (CD31) of wound tissue sections on day 21 (scale bar = 50 µm, red circle is immunofluorescence staining of CD31). (**B**) CD31-positive area of wound tissue sections on day 21 (*n* = 3). (**C**) The collagen volume fraction of wound tissue sections on day 21 (*n* = 3). Data represent mean ± SD; * *p* < 0.05, ** *p* < 0.01, *** *p* < 0.001.

To assess the effect of the CS/rCol/Cu_100_/DMA hydrogel on tissue regeneration at the wound site, hematoxylin and eosin (H & E) staining and Masson trichrome staining of the wound skin were performed on day 21. The H & E staining results of the wounds (Figure 10A) for the negative and positive control groups showed inflammatory cell infiltration, granulation tissue formation, neovascularization, and an increase in fibroblast migration. However, the wounds in the CS/rCol/Cu_100_/DMA hydrogel group exhibited extensive near-mature neovascularization and granulation tissue formation and contained a high number of fibroblasts that were closely arranged on the wound surface. Taken together, these effects demonstrate that the hydrogel possesses a strong skin regeneration ability (Figure 10A). In addition, collagen deposition was assessed using Masson trichrome staining. As shown in Figure 10B, in contrast to the negative and positive control groups, the hydrogel-treated group showed denser and more regular collagen fibers (i.e., blue-labeled fibers are collagen fibers) by day 21. A quantitative analysis of collagen density on day 21 revealed that the hydrogel group had a higher collagen content (66.96%) than the negative control group (29.3%) or the positive control group (45.1%) (Figure 11C). These results further confirm the efficient wound-healing ability of the hydrogel.

Finally, angiogenesis is essential for tissue regeneration and wound healing. Platelet endothelial cell adhesion molecule-1 (CD31) is a hallmark of vascular endothelial cell differentiation [63]. Therefore, red immunofluorescence dye was used to label CD31 and thereby to track angiogenesis. Figure 11A shows the CD31 staining images for different treatment groups (red circle in Figure 11A). The hydrogel group had noticeably newer and more mature blood vessels by day 21 than the other groups. Quantitative findings showed that the CD31 density of the hydrogel group was the highest of all groups on day 21 (Figure 11B). Taken together, these results highlight that the biological macromolecule hydrogel can effectively adhere to wounds, promote tissue regeneration, inhibit bacterial growth, and stimulate wound healing in vivo.

## 4. Conclusions

In conclusion, a multifunctional, biological macromolecule hydrogel based on a recombinant type I collagen/chitosan scaffold incorporated with a metal–polyphenol structure was developed to accelerate wound healing. The dual-network structure endows this hydrogel with sufficient mechanical strength and many functions useful for the effective treatment of open wounds, including self-healing, adhesiveness, and antibacterial, antioxidant, and provascularization abilities. Moreover, the multifunctional, biological macromolecule hydrogel also shows high biocompatibility and hemostatic properties. The CS/rCol/Cu/DMA hydrogel was found to promote acute wound healing in vivo by facilitating granulation tissue formation and collagen deposition. Therefore, this hydrogel could successfully satisfy critical requirements at different stages of the wound repair process. For example, in the early stage of wound healing, the hydrogel exhibited rapid hemostasis and antibacterial effects. In the middle and later stages of wound healing, the hydrogel could reduce wound reactive oxygen species and stimulate vascularization and collagen deposition. Taken together, these results demonstrate that the multifunctional, biological macromolecule hydrogel can serve as a reliable dressing agent for clinical treatments. We believe further research could be performed on this collagen-based multifunctional composite in the future, especially with the addition of some single components, including different Chinese herb polysaccharides, which could lead to unexpected surprises.

## Data Availability

The data that support the findings of this study are available on request from the corresponding author.

## Supplementary Materials

The following supporting information can be downloaded at: https://www.mdpi.com/article/10.3390/polym15193919/s1, References [34,46,49,51,54,64,65,66,67] are cited in the Supplementary Materials.
