# Supplementary material for: Biological Macromolecule Hydrogel Based on Recombinant Type I Collagen/Chitosan Scaffold to Accelerate Full-Thickness Healing of Skin Wounds
Source: Polymers (Basel). 2023 Sep 28;15(19):3919. doi: 10.3390/polym15193919 (PMC10575414; doi:10.3390/polym15193919)
Supplement: Supplementary file 1 [file polymers-15-03919-s001.zip › polymers-2581911-supplementary.pdf]

Supplementary Information

Biological macromolecule hydrogel based on recombinant type I collagen/chitosan scaffold to accelerate full-thickness healing of skin wounds

S1. Supplementary materials and methods

S1.1 Materials

Dopamine hydrochloride (DA) (98%), sodium tetraborate (99%), methacrylic anhydride (94%), and magnesium sulfate anhydrous (99%) were purchased from MACKLIN (Shanghai, China). Tetrahydrofuran (99.5%), deuterium oxide (99.9%), dimethylsulfoxide-d6 (DMSO-d6) (99.8%), glycidyl methacrylate (98%), and cupric chloride (98%) were obtained from Energy Chemical (Shanghai, China). Chitosan (CS) (low molecular weight) was acquired from Sigma-Aldrich (St. Louis, Missouri, USA). Dulbecco's modified Eagle's medium (DMEM) and Cell Counting Kit-8 (CCK-8) were purchased from Gibico (California, USA) and Beyotime (Shanghai, China), respectively. Copper Assay Kit was purchased from MesGen® Biotechnology (Shanghai, China).

S1.2 Recombinant type I collagen sequence

S1.2.1 Amino acid sequence

(GERGDLGPQGIAGQRGVVGERGERGERGASGFPGER）$_8$ GGGS (GERGDLGPQGIAGQRGVVGERGERGERGASGFPGER）$_8$ GGGS (GERGDLGPQGIAGQRGVVGERGERGERGASGFPGER）$_8$ GGGS (GERGDLGPQGIAGQRGVVGERGERGERGASGFPGER）$_8$

S1.2.2 Nucleotide sequence

ATGGGCGAGCGCGGCGACTTAGGCCCG-
CAAGGCATCGCGGGTCAGCGTGGCGTAGTT-
GGTGAACGGGGTGAACGGGGTGAGCGTGGTGCTAGCGGCTTTCCGGGTGAACGT
GGGGAACGTGGTGATCTGGGCCCGCAGGGTATCGCGGGTCAGCGTGGTGTT-
GTGGGCGAAC-
GTGGTGAACGTGGTGAGCGCGGTGCATCGGGCTTTCCAGGCGAACGCGGGGAA
CGCGGTGATCTGGGGCCGCAGGGTATTGCCGGCCAACGTGGCGTGGTT-
GGTGAGCGGGGTGAGCGCGGCGAGCGCGGTGCGAGCGGTTTCCCCGGCGAACG
TGGCGAGCGTGGCGACCTGGGCCCG-
CAAGGCATCGCTGGCCAGCGTGGCGTGGTCGGTGAAC-
GTGGCGAACGTGGGGAACGTGGCGCCTCTGGCTTCCCTGGCGAACGAGGTGAAC
GTGGGGATCTGGGCCCGCAAGGAATTGCGGGTCAGCGCGGCGTTGTT-
GGCGAACGCGGA-
GAGCGCGGTGAGCGTGGCGCGAGCGGCTTTCCGGGCGAACGCGGCGAACGCGG
TGATCTGGGTCCGCAGGGCATCGCTGGTCAACGTGGCGTAGTCGGTGAGCGTG-
GAGAAC-
GTGGCGAACGTGGCGCGTCCGGCTTTCCCGGTGAACGAGGCGAACGTGGGGAC
CTGGGTCCACAGGGTATCGCTGGCCAGCGCGGCGTTGTGGGA-
GAGCGTGGCGAAC-
GCGGCGAACGCGGTGCATCTGGCTTCCCAGGCGAACGTGGAGAACGCGGTGAC
CTGGGTCCGCAGGG-
CATCGCGGGCCAGCGCGGTGTGGTCGGCGAGCGTGGTGAAC-
GTGGTGAACGGGGCGCGTCCGGTTTCCCAGGTGAAAGAGGTGGCGGTTCCGGAT

CCGGCGAGCGCGGCGACTTAGGCCCGCAAGGCATCGCGGGTCAGCGTGGCG-
TAGTT-
GGTGAACGGGGTGAACGGGGTGAGCGTGGTGCTAGCGGCTTTCCGGGTGAACGT
GGGGAACGTGGTGATCTGGGCCCGCAGGGTATCGCGGGTCAGCGTGGTGTT-
GTGGGCGAAC-
GTGGTGAACGTGGTGAGCGCGGTGCATCGGGCTTTCCAGGCGAACGCGGGGAA
CGCGGTGATCTGGGGCCGCAGGGTATTGCCGGCCAACGTGGCGTGGTT-
GGTGAGCGGGGTGAGCGCGGCGAGCGCGGTGCGAGCGGTTTCCCCGGCGAACG
TGGCGAGCGTGGCGACCTGGGCCCG-
CAAGGCATCGCTGGCCAGCGTGGCGTGGTCGGTGAAC-
GTGGCGAACGTGGGGAACGTGGCGCCTCTGGCTTCCCTGGCGAACGAGGTGAAC
GTGGGGATCTGGGCCCGCAAGGAATTGCGGGTCAGCGCGGCGTTGTT-
GGCGAACGCGGA-
GAGCGCGGTGAGCGTGGCGCGAGCGGCTTTCCGGGCGAACGCGGCGAACGCGG
TGATCTGGGTCCGCAGGGCATCGCTGGTCAACGTGGCGTAGTCGGTGAGCGTG-
GAGAAC-
GTGGCGAACGTGGCGCGTCCGGCTTTCCCGGTGAACGAGGCGAACGTGGGGAC
CTGGGTCCACAGGGTATCGCTGGCCAGCGCGGCGTTGTGGGA-
GAGCGTGGCGAAC-
GCGGCGAACGCGGTGCATCTGGCTTCCCAGGCGAACGTGGAGAACGCGGTGAC
CTGGGTCCGCAGGG-
CATCGCGGGCCAGCGCGGTGTGGTCGGCGAGCGTGGTGAAC-
GTGGTGAACGGGGCGCGTCCGGTTTCCCAGGTGAAAGAGGTGGCGGTTCCGAGC
TCGGCGAGCGCGGCGACTTAGGCCCGCAAGGCATCGCGGGTCAGCGTGGCG-
TAGTT-
GGTGAACGGGGTGAACGGGGTGAGCGTGGTGCTAGCGGCTTTCCGGGTGAACGT
GGGGAACGTGGTGATCTGGGCCCGCAGGGTATCGCGGGTCAGCGTGGTGTT-
GTGGGCGAAC-
GTGGTGAACGTGGTGAGCGCGGTGCATCGGGCTTTCCAGGCGAACGCGGGGAA
CGCGGTGATCTGGGGCCGCAGGGTATTGCCGGCCAACGTGGCGTGGTT-
GGTGAGCGGGGTGAGCGCGGCGAGCGCGGTGCGAGCGGTTTCCCCGGCGAACG
TGGCGAGCGTGGCGACCTGGGCCCG-
CAAGGCATCGCTGGCCAGCGTGGCGTGGTCGGTGAAC-
GTGGCGAACGTGGGGAACGTGGCGCCTCTGGCTTCCCTGGCGAACGAGGTGAAC
GTGGGGATCTGGGCCCGCAAGGAATTGCGGGTCAGCGCGGCGTTGTT-
GGCGAACGCGGA-
GAGCGCGGTGAGCGTGGCGCGAGCGGCTTTCCGGGCGAACGCGGCGAACGCGG
TGATCTGGGTCCGCAGGGCATCGCTGGTCAACGTGGCGTAGTCGGTGAGCGTG-
GAGAAC-
GTGGCGAACGTGGCGCGTCCGGCTTTCCCGGTGAACGAGGCGAACGTGGGGAC
CTGGGTCCACAGGGTATCGCTGGCCAGCGCGGCGTTGTGGGA-
GAGCGTGGCGAAC-
GCGGCGAACGCGGTGCATCTGGCTTCCCAGGCGAACGTGGAGAACGCGGTGAC
CTGGGTCCGCAGGG-
CATCGCGGGCCAGCGCGGTGTGGTCGGCGAGCGTGGTGAAC-
GTGGTGAACGGGGCGCGTCCGGTTTCCCAGGTGAAAGAGAATTCGGTAGCGGCA
GTGGTTCTGGCGAGCGCGGCGACTTAGGCCCG-
CAAGGCATCGCGGGTCAGCGTGGCGTAGTT-
GGTGAACGGGGTGAACGGGGTGAGCGTGGTGCTAGCGGCTTTCCGGGTGAACGT
GGGGAACGTGGTGATCTGGGCCCGCAGGGTATCGCGGGTCAGCGTGGTGTT-
GTGGGCGAAC-
GTGGTGAACGTGGTGAGCGCGGTGCATCGGGCTTTCCAGGCGAACGCGGGGAA
CGCGGTGATCTGGGGCCGCAGGGTATTGCCGGCCAACGTGGCGTGGTT-
GGTGAGCGGGGTGAGCGCGGCGAGCGCGGTGCGAGCGGTTTCCCCGGCGAACG

TGGCGAGCGTGGCGACCTGGGCCCG-
CAAGGCATCGCTGGCCAGCGTGGCGTGGTCGGTGAAC-
GTGGCGAACGTGGGGAACGTGGCGCCTCTGGCTTCCCTGGCGAACGAGGTGAAC
GTGGGGATCTGGGCCCGCAAGGAATTGCGGGTCAGCGCGGCGTTGTT-
GGCGAACGCGGA-
GAGCGCGGTGAGCGTGGCGCGAGCGGCTTTCCGGGCGAACGCGGCGAACGCGG
TGATCTGGGTCCGCAGGGCATCGCTGGTCAACGTGGCGTAGTCGGTGAGCGTG-
GAGAAC-
GTGGCGAACGTGGCGCGTCCGGCTTTCCCGGTGAACGAGGCGAACGTGGGGAC
CTGGGTCCACAGGGTATCGCTGGCCAGCGCGGCGTTGTGGGA-
GAGCGTGGCGAAC-
GCGGCGAACGCGGTGCATCTGGCTTCCCAGGCGAACGTGGAGAACGCGGTGAC
CTGGGTCCGCAGGG-
CATCGCGGGCCAGCGCGGTGTGGTCGGCGAGCGTGGTGAAC-
GTGGTGAACGGGGCGCGTCCGGTTTCCCAGGTGAAAGATAA

S1.3 Synthesis of recombinant type I collagen

Genetic engineering technique was used to produce recombinant collagen [1]. The specific experimental process is shown in Figure S2A.

S1.4 Biocompatibility test of recombinant type I collagen

The NIH/3T3 cells were seeded in a 96-well plate with 5 × 10³ cells/well. After cell adhesion, the DMEM medium was replaced with an rCol solution (100 μL). NIH/3T3 cells were cultured in an incubator for 24 h and then tested at 450 nm according to the instructions of the CCK-8 kit. The cell viability was as follows:

$$\text{Cell viability (\%)} = \frac{\text{experimental group} - \text{blank group}}{\text{control group} - \text{blank group}} \times 100\%$$

S1.5 Cell adhesion test of recombinant type I collagen

High (100 μg mL^{-1}), medium (50 μg mL^{-1}), and low (12.5 μg mL^{-1}) rCol concentrations were selected and evenly spread in a 96-well plate without TC treatment and then dried to form an rCol membrane. The NIH/3T3 cell suspension was seeded at a density of 5×10^3 cells/well in the 96-well plate with an rCol membrane and cultured for 4 h. Then, absorbance was measured at 450 nm using a CCK-8 kit. Wells without an rCol membrane were used as a control.

S1.6 Synthesis of DMA

Methacrylamide dopamine (DMA) was synthesized using the reported method [2]. First, 20 g of sodium tetraborate and 8 g of sodium bicarbonate were dissolved in 200 mL of deionized water and bubbled with nitrogen for 60 minutes. Then, 10 g of dopamine hydrochloride was added to the mixture and stirred under nitrogen to obtain a dopamine solution. Fifty mL of the deoxygenated tetrahydrofuran solution was taken and 9.4 mL of methacrylate anhydride was added to obtain an acrylic anhydride solution. The methacrylate anhydride solution was gradually added dropwise to the dopamine solution, and the pH was adjusted by adding 1.0 M NaOH (after deoxygenation). The pH was periodically maintained at 8-9. After stirring at room temperature for 17 h, the solution was extracted once with ethyl acetate (50 mL), and the aqueous layer was retained; then, the pH was adjusted to 2 with 6 M HCl. The mixture was extracted twice with ethyl acetate (50 mL), and the organic layer was retained. After that, the mixture was dried with anhydrous magnesium sulfate and distilled under reduced pressure to obtain the product as dopamine methacrylate (DMA).

S1.7 Synthesis of CSMA

Methacrylate chitosan (CSMA) was synthesized according to the previously described method [3, 4]. Briefly, 3% (w/v) chitosan powder was dissolved in a 3% (w/v) acetic acid solution at room temperature for 24 h. Methacrylate was added to the chitosan solution at a ratio of 3.5:1 (w/w) and reacted at room temperature for 3 h. The reaction mixture was dialyzed (in a 14 kDa dialysis bag) for 6 days. The resultant mixture was freeze-dried and stored at -20 °C for further use.

S1.8 Synthesis of rCol-GMA

The synthesis of glycidyl methacrylate recombinant collagen (rCol-GMA) is similar to that of silk fibroin [5]. Recombinant type I collagen (120 kDa) (10% w/v) was dissolved in PBS solution (100 mL) at 4 °C until it was completely dissolved. Then, glycidyl methacrylate (GMA) was added at a collagen–glycidyl methacrylate ratio of 1:0.6. The mixed solution was stirred for 8 h until it turned milky white at 4 °C. Finally, the mixed solution was dialyzed in deionized water at 4 °C by using a dialysis bag (14 kDa) for 5 days. The resultant mixture was freeze-dried and stored at -20 °C for further use.

S1.9 Characterizations

The FT-IR spectra of DA, DMA, CS, CSMA, rCol, and rCol-GMA were recorded on an iS50 FT-IR spectrometer (Thermo Scientific, California, USA) in the 4000–500 cm^{-1} range.

The H nuclear magnetic resonance (^{1}H-NMR) (Ascend 400, Bruker, Luken, Germany) of rCol and rCol-GMA was recorded at 25 °C using D_2O as a solvent; CS and CSMA were recorded at 25 °C using D_2O with 1% trifluoroacetic acid as a solvent; DA and DMA were recorded at 25 °C using DMSO-d6 as a solvent.

The morphology of hydrogels was examined using a field emission scanning electron microscope (SEM; FEI NOVA NANO450, Columbia, Maryland, USA) after all the hydrogel samples were freeze-dried and sprayed with a thin gold layer.

S1.10 Swelling test

The freeze-dried hydrogel (500 μL) was immersed in 2 mL PBS at room temperature. The hydrogel was taken out of the PBS at each predetermined time interval, and excess fluid was removed with filter paper. The calculation formula for the swelling ratio was as follows: Swelling ratio (%) = $\frac{(W_t - W_0)}{W_0} \times 100\%$

W_0 and W_t are the initial weight of the hydrogel after freeze-drying and the weight after swelling, respectively.

S1.11 Rheological, shear-thinning, and shelf-healing property test

The rheological measurements of the hydrogels were tested using a TA rheometer instrument (HR30, WATERS, Newcastle, Delaware, USA). A plate–plate geometry (8 mm diameter, 500 μm gap) was used for all measurements. Strain amplitude sweeps were performed under a strain of 0% to 500% with a fixed frequency of 10 rad s^{-1}. The viscosity of the hydrogel was also analyzed at different shear rates (0.01–100 s^{-1}).

The self-healing performance of hydrogels was measured by macroscopic observations and quantitative methods. In microscopic observations, CS/rCol/Cu_{100}/DMA hydrogel was used as a representative. The hydrogel was cut into halves, and one piece of cylinder-shaped hydrogel was stained with purple. Then, cuts of each piece were put together at about 5 min without any external stimuli at room temperature.

The self-healing performance was also confirmed using the rheometer. To investigate the structure recovery ability of the hydrogel, the time sweep was carried out at room temperature by measuring the G′ and G″ data with time at alternating low stress (in the linear viscoelastic region) and high stress (beyond the linear viscoelastic region).

S1.12 Compression test of CS/rCol/Cu/DMA hydrogels

The compression test was according to the previous report [6]. The compressive stress–strain measurements were performed using a Universal testing machine (DMA Q800, TA, Newcastle, Delaware, USA). For the test, a cylindrical probe with 25 mm was used to compress hydrogels. The ramp force was 1.0000 N min^{-1} to 3.0000 N. The successive loading–unloading compressive tests were measured at different times. All the tests were performed at 25 °C.

S1.13 The release kinetics of copper ions in vitro

The Copper Assay Kit (MesGen® Biotechnology, Shanghai, China) was used to measure the total content of copper ions in the hydrogel. The release behavior of copper ions was also detected in vitro. Briefly, 500 μL of hydrogel was incubated in 2 mL PBS with shaking (150 rpm, 37 °C) for 3 days. At different time intervals (15 min, 30 min, 45 min, 60 min, 75 min, 90 min, 105 min, 120 min, 135 min, 150 min, 165 min, 180 min, 3.5 h, 4.5 h, 5.5 h, 7.5 h, 11.5 h, 24 h, 72 h, 84 h), 0.5 mL of supernatant was taken and an equal volume of fresh solution was added for replenishment. The supernatant was detected according to the manufacturer's instructions.

S1.14 Biocompatibility test in vitro

HUVECs were used to evaluate the cytocompatibility of the hydrogel. The biocompatibility of the hydrogel was determined by using the hydrogel extract method and CCK-8 assays. The hydrogel extract liquid was obtained by adding 5 mL of endothelial cell medium (ECM) to a 0.5 g hydrogel and then incubated in an incubator (37 °C, 5% CO_2) for 24 h. The HUVECs were firstly cultured in ECM for 24 h and then seeded in 96-well microtiter plates at a density of 5×10^4 cells mL^{-1}. After incubating for 12 h, the medium was replaced by a fresh medium containing a different hydrogel extract liquid. After incubation of the cells with different hydrogel extract liquids for 1, 2, and 3 days, the medium was replaced by fresh CCK-8 solution (10 μL CCK-8 + 90 μL medium) and cultured for 1–3 h, which was followed by measurement of absorbance at 450 nm using a microplate reader. The cell viability was as follows:

$$\text{Cell viability (\%)} = \frac{\text{experimental group} - \text{blank group}}{\text{control group} - \text{blank group}} \times 100\%$$

S1.15 ROS scavenging ability in vitro

NIH/3T3 cells were seeded at a density of 5×10^3 cells per well in a 96-well plate and pretreated with PMA (150 μg mL^{-1}) to elevate the intracellular ROS level, followed by treatment with different hydrogel extract solutions for 4 h. Dichlorofluorescein diacetate (DCFH-DA) was employed to detect the intracellular ROS level. Finally, the fluorescence signal was measured with a fluorescence microplate. Fluorescence was read using 488 nm as the excitation wavelength and 525 nm as the emission wavelength. The remaining intracellular ROS was as follows:

$$\text{Remaining intracellular ROS (\%)} = \frac{\text{experimental group}}{\text{PMA treated control group}} \times 100\%$$

S1.16 Angiogenesis test

The angiogenesis of the hydrogel was evaluated according to a previous study [7]. Briefly, Matrigel and 96-well plates were thawed at 4 °C overnight. Afterward, Matrigel (50 μL) was dropped into precooled 96-well plates and then placed statically at 37 °C for 1 h. Subsequently, HUVECs (100 μL) were seeded in each well at a density of 5×10^3 cells/well, and the different hydrogel extract solutions were added at the same time. After incubation for 4 h, the angiogenic capacity of the hydrogel on HUVECs was recorded by photographing with a mobile phone.

S1.17 Hemostasis performance of hydrogels

Referring to previous research [8], the hemostatic ability of hydrogels was evaluated in rat hepatic trauma models (SD, 180-200 g, female). All animal studies were approved by the Institutional Animal Care and Use Committee of the South China University of Technology (Approval No. 2019053). Animals were anesthetized by inhalation of isoflurane and fixed on a 30° inclined operating table. Mouse livers were exposed through abdominal incisions, and serous fluid around the liver was carefully removed to prevent inaccurate estimates of blood weight obtained from hemostatic samples. Filter paper lined with plastic sheeting was placed under the liver. A needle was then used to cause the liver to bleed. Afterward, the hydrogel was applied to the bleeding area. The liver was punctured without treatment as a control group. After 1 minute, the weight of the filter paper absorbing blood was recorded. The blood loss was as follows:

Blood loss (mg) = (weight of filter paper after blood sucking-Original filter paper weight)

S1.18 Establishment of full-layer skin defect model in vivo

All mice were divided into 3 groups: control, positive control (Extra Thin CGF™ Dressing, CONVATEC), and CS/rCol/Cu_{100}/DMA hydrogel. All mice were anesthetized with isoflurane inhalation. The backs of the mice were shaved and depilated with hair removal cream (Veet). Then, 2 cm diameter, full-thickness skin round wounds were created using a coin. After the removal of wound skin, positive control group wounds were dressed with Extra Thin CGF™ Dressing, and hydrogel group wounds were dressed with CS/rCol/Cu_{100}/DMA hydrogel.

S1.19 Histological examination and immunohistochemistry

Histological examination: Firstly, fresh skin tissues cut from the wounds of SD rats were fixed in a fixative solution for 24 h. The tissues were removed and repaired with a scalpel in a fume hood and then dehydrated in an automatic dehydrator.

Secondly, the dehydrated tissue was removed from the dehydrator for embedding. Melted wax was poured into the embedding frame. The tissue was then placed for embedding.

Thirdly, the wax blocks were placed on a freezing table at -20 °C. The frozen wax was clamped onto a paraffin slicer and cut into 4-6 micro slices, after which the slices were placed on the water surface of the slicer (water temperature 40 °C). Then, the slices were picked up with a slide. Finally, the slices were placed in an oven at 60 °C for 1 hour to be baked for other experiments (e.g., H&E and Masson)

Immunohistochemical methods: Samples were embedded and sectioned. Sections were incubated with 30% normal sheep serum diluted in PBS at 25 °C for 1 h. Then, sections were incubated with rabbit anti-CD31 primary antibody (Abcam, ab28364, 1:500) and washed with PBS, and then the secondary antibody (Abcam, ab205719, 1:1000) was used. Images of stained samples were captured using an inverted fluorescence microscope (TE2000-S, Nikon, Tokyo, Japan). Image J 1.52v (Wayne Rasband National Institutes of Health, USA)[9] software was used to analyze the collected images and calculate the percentage of positive markers.

Table S1. Sample parameters of hydrogels.

Sample	CSMA (4 wt%)	rCol-GMA (4 wt%)	DMA (8.225 wt%)	Cu^{2+} (10 wt%)	LAP (1 wt%)	DI water	Gelation time
CS/rCol	400 µL	400 µL	0 µL	0 µL	50 µL	150 µL	15 s
CS/rCol/Cu_{50}/DMA	400 µL	400 µL	2 µL	0.5 µL	50 µL	147.5 µL	15 s
CS/rCol/Cu_{100}/DMA	400 µL	400 µL	4 µL	1 µL	50 µL	145 µL	15 s
CS/rCol/Cu_{200}/DMA	400 µL	400 µL	8 µL	2 µL	50 µL	140 µL	15 s

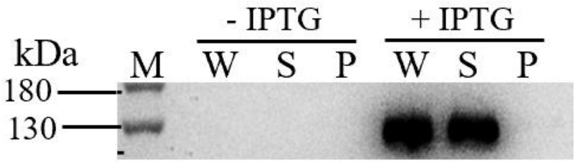

Figure S1. Western blot of recombinant collagen (rCol) (M: protein marker (Thermo Fisher #26616); W: whole cell lysates; S: supernatant; P: precipitate).

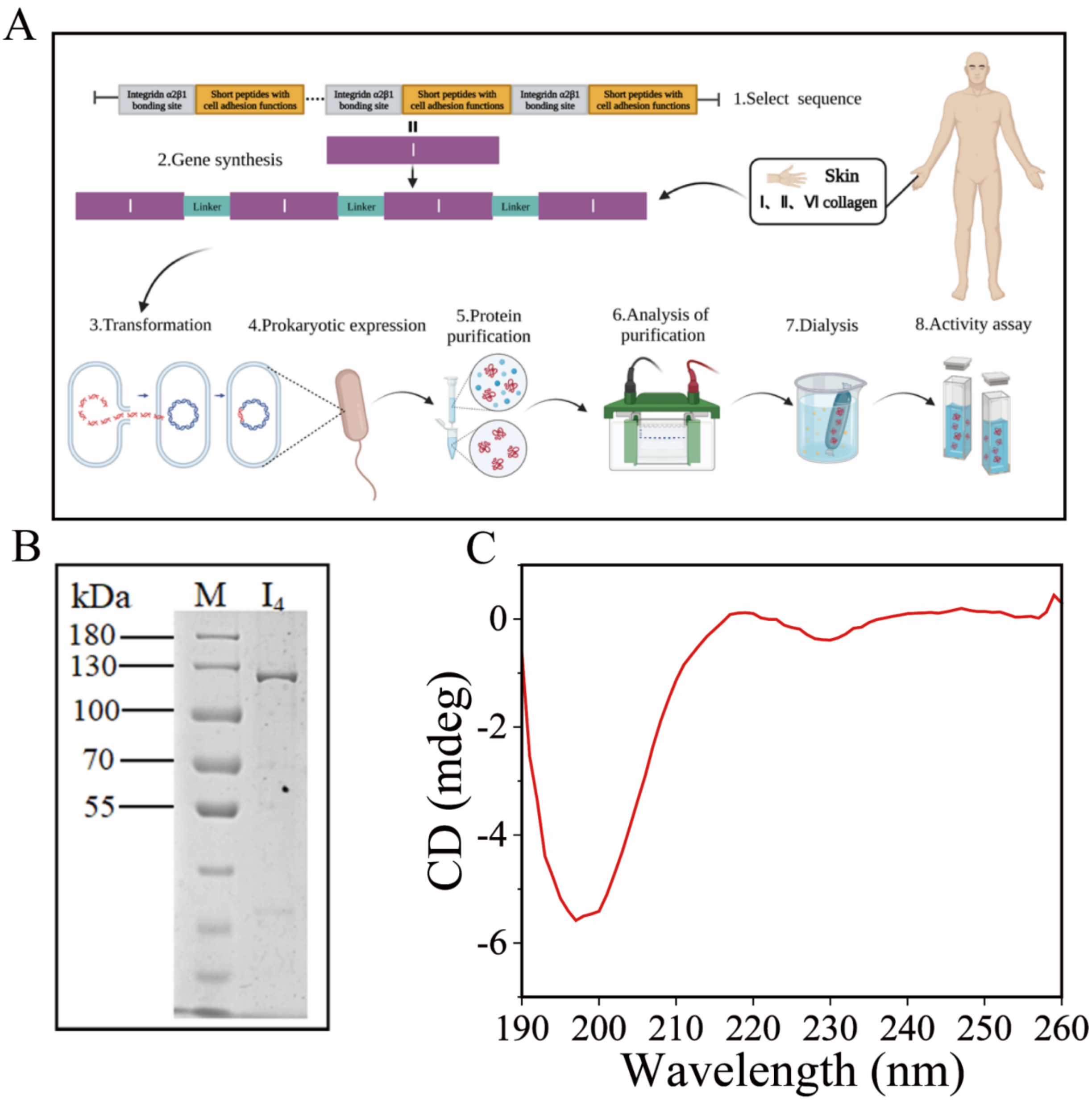

Figure S2. (A) Schematic diagram of the synthesis of rCol. (B) Sodium dodecyl sulfate polyacrylamide gel electrophoresis (SDS-PAGE) of rCol after purification. (C) Circular dichroic spectrum (CD) of rCol.

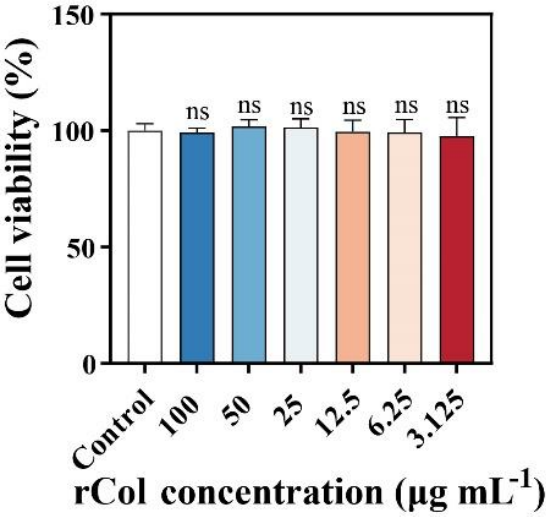

Figure S3. Cell viability of NIT/3T3 cells cultured in an rCol solution using a CCK-8 assay (n = 3). Data represent mean ± SD; **p* < 0.05, ***p* < 0.01, ****p* < 0.001.

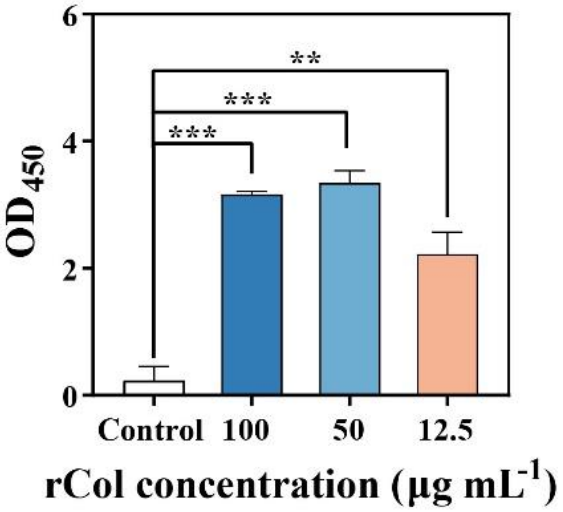

Figure S4. Cell adhesion of NIT/3T3 cells cultured in an rCol membrane using a CCK-8 assay (n = 3). Data represent mean ± SD; **p* < 0.05, ***p* < 0.01, ****p* < 0.001.

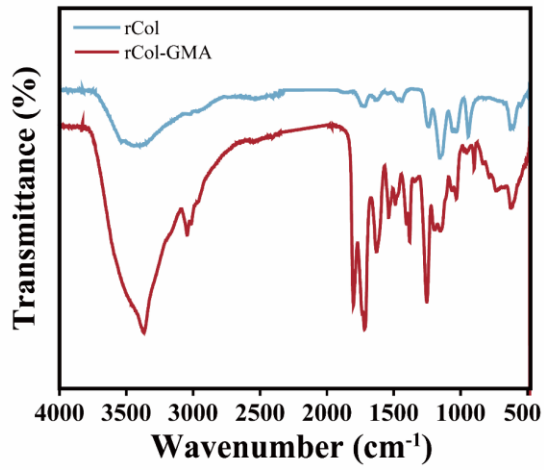

Figure S5. Fourier transform infrared spectroscopy (FT-IR) spectra of rCol and rCol-GMA.

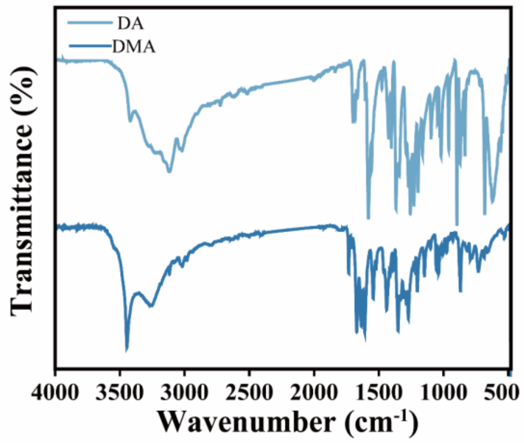

Figure S6. Fourier transform infrared spectroscopy (FT-IR) spectra of DA and DMA.

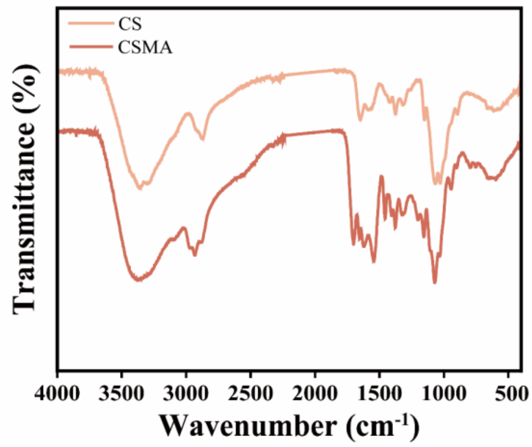

Figure S7. Fourier transform infrared spectroscopy (FT-IR) spectra of CS and CSMA.

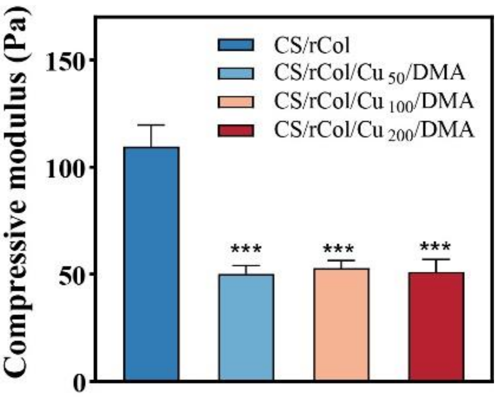

Figure S8. The compressive modulus of hydrogels (n = 3). Data represent mean ± SD; $*p < 0.05$, $**p < 0.01$, $***p < 0.001$.

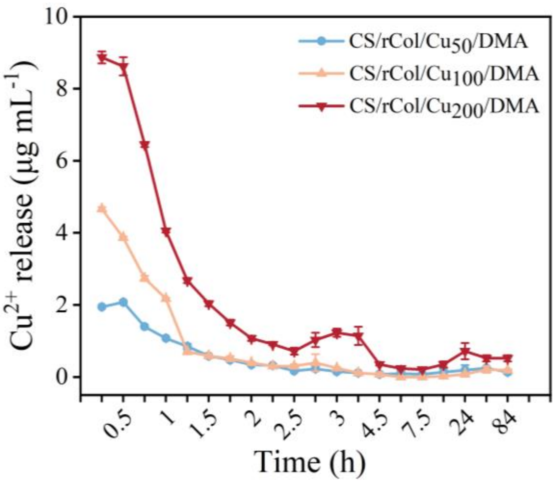

Figure S9. Cu^{2+} release from hydrogels (n = 3). Data represent mean ± SD; $*p < 0.05$, $**p < 0.01$, $***p < 0.001$.

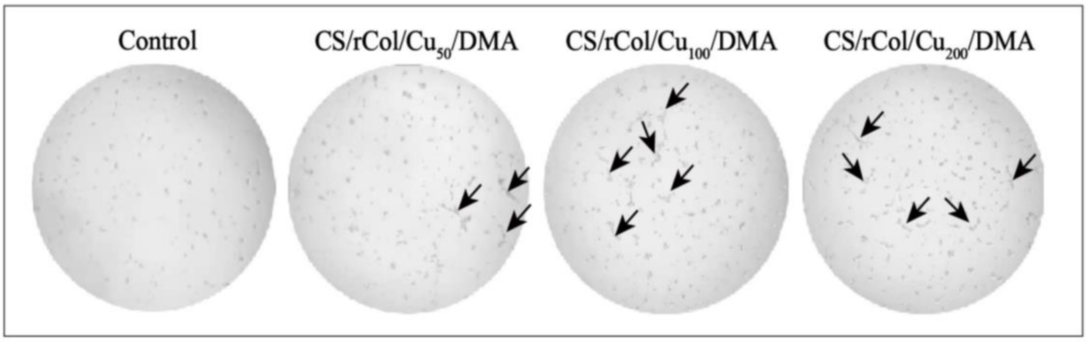

Figure S10. Photographs of in vitro tube formation in human umbilical vein endothelial cells (HUVECs) cultured with the hydrogel extract solution (microscope eyepiece is 10 ×, objective lens is 10 ×, black arrows indicate the formed tubes).

Reference

1. Liu, S.; Li, Y.; Wang, M.; Ma, Y.; Wang, J., Efficient coexpression of recombinant human fusion collagen with prolyl 4-hydroxylase from Bacillus anthracis in Escherichia coli. *Biotechnol Appl Biochem* **2022**.
2. Hou, S.; Ma, P. X., Stimuli-responsive supramolecular hydrogels with high extensibility and fast self-healing via precoordinated mussel-inspired chemistry. *Chem Mater* **2015,** *27* (22), 7627-7635.
3. He, X.; Liu, X.; Yang, J.; Du, H.; Chai, N.; Sha, Z.; Geng, M.; Zhou, X.; He, C., Tannic acid-reinforced methacrylated chitosan/methacrylated silk fibroin hydrogels with multifunctionality for accelerating wound healing. *Carbohydr Polym* **2020,** *247*, 116689.

4. Bozuyuk, U.; Yasa, O.; Yasa, I. C.; Ceylan, H.; Kizilel, S.; Sitti, M., Light-Triggered Drug Release from 3D-Printed Magnetic Chitosan Microswimmers. *ACS Nano* **2018,** *12* (9), 9617-9625.
5. Lu, K.; Li, K. K.; Zhang, M.; Fang, Z. X.; Wu, P. P.; Feng, L. B.; Deng, K. X.; Yu, C. J.; Deng, Y. Z.; Xiao, Y.; Zhu, P.; Guo, R., Adipose-derived stem cells (ADSCs) and platelet-rich plasma (PRP) loaded gelatin/silk fibroin hydrogels for improving healing in a murine pressure ulcer model. *Chemical Engineering Journal* **2021,** *424*.
6. Zhang, B.; He, J.; Shi, M.; Liang, Y.; Guo, B., Injectable self-healing supramolecular hydrogels with conductivity and photo-thermal antibacterial activity to enhance complete skin regeneration. *Chemical Engineering Journal* **2020,** *400*.
7. Zhou, L.; Liu, N.; Feng, L.; Zhao, M.; Wu, P.; Chai, Y.; Liu, J.; Zhu, P.; Guo, R., Multifunctional electrospun asymmetric wettable membrane containing black phosphorus/Rg1 for enhancing infected wound healing. *Bioeng Transl Med* **2022,** *7* (2), e10274.
8. Yang, Y.; Liang, Y.; Chen, J.; Duan, X.; Guo, B., Mussel-inspired adhesive antioxidant antibacterial hemostatic composite hydrogel wound dressing via photo-polymerization for infected skin wound healing. *Bioact Mater* **2022,** *8*, 341-354.
9. Rueden, C. T.; Schindelin, J.; Hiner, M. C.; DeZonia, B. E.; Walter, A. E.; Arena, E. T.; Eliceiri, K. W., ImageJ2: ImageJ for the next generation of scientific image data. *BMC Bioinformatics* **2017,** *18* (1), 529.
